# Mechanical and Thermal Properties of W-Ta-B Coatings Deposited by High-Power Impulse Magnetron Sputtering (HiPIMS)

**DOI:** 10.3390/ma16020664

**Published:** 2023-01-10

**Authors:** Rafał Psiuk, Tomasz Mościcki, Justyna Chrzanowska-Giżyńska, Łukasz Kurpaska, Joanna Radziejewska, Piotr Denis, Dariusz Garbiec, Marcin Chmielewski

**Affiliations:** 1 Institute of Fundamental Technological Research, Polish Academy of Sciences, Pawinskiego 5B, 02-106 Warsaw, Poland; 2 NOMATEN Centre of Excellence, National Centre for Nuclear Research, ul. A. Soltana 7, 05-400 Otwock, Poland; 3 Faculty of Mechanical and Industrial Engineering, Warsaw University of Technology, Narbutta 85, 02-524 Warsaw, Poland; 4 Łukasiewicz Research Network – Poznań Institute of Technology, 6 Ewarysta Estkowskiego St., 61-755 Poznan, Poland; 5 Łukasiewicz Research Network – Institute of Microelectronics and Photonics, Centre of Functional Materials, 133 Wolczynska St., 01-919 Warsaw, Poland

**Keywords:** tungsten diboride, high power impulse magnetron sputtering (HiPIMS), hardness, thermal stability, oxidation resistance

## Abstract

We present the deposition and characterization of tungsten-tantalum diboride (W,Ta)B_2_ coatings prepared by the high-power impulse magnetron sputtering technique. We evaluated the influence of pulse duration and substrate bias on the properties of (W,Ta)B_2_ films. A high hardness of up to 35 GPa measured by nanoindentation was simultaneously obtained with good elastic properties. Changing the pulse duration greatly affected the B/(W+Ta) atomic ratio, which influenced the properties of the coatings. The deposited films are thermally stable at up to 1000 °C in vacuum and are able to withstand oxidation at 500 °C.

## 1. Introduction

Superhard materials are extensively used in modern industries. Diamond and cubic boron nitride are the most popular, yet they possess many drawbacks, such as a high affinity to iron (diamond), high pressure synthesis (diamond, c-BN), and lesser adhesion of films in humid atmospheres (c-BN). These are among the motives for research on new superhard materials.

Transition metal borides can be an alternative to traditional superhard materials in many applications. They can be superhard, have good electrical and thermal conductivity, and usually possess a high melting point [1,2,3]. In addition, high pressure is not needed to synthesize them. Within this class of materials, tungsten borides are considered to be low-cost superhard materials. While WB_4_ is intrinsically superhard, WB_2_ can be extrinsically hardened. The most popular ways to obtain superhard WB_2_ are grain refinement or solid solutions with other transition metals [4]. Moreover, tungsten borides have good fracture toughness in comparison to nitrides such as TiN, TiAlN, or ZrN, which are typically used as coating materials on tools [5].

Alloying by transition metals can significantly enhance the properties of tungsten borides. The addition of titanium [6] leads to an improvement of the tribological properties of the coating. In addition, (W,Ti)B_2_ coatings have shown higher corrosion resistance than unalloyed WB_2_. The addition of zirconium [7] leads to an increase in the thermal stability without affecting the mechanical properties. Fuger et al. [5] show that the addition of tantalum can increase the hardness up to 45 GPa while only lowering the fracture toughness to 3 MPa × m^1/2^, which is higher than for TiN coatings. In that paper, the deposition of (W,Ta)B_2_ was performed by DC magnetron sputtering with the use of W_2_B_5_ and TaB_2_ targets. Furthermore, 700 °C [5] was used as a substrate temperature, which is much higher than the temperature used in the present study.

High-power impulse magnetron sputtering (HiPIMS) employs short pulses (a few to tens of microseconds) with exceptionally high power density. Such conditions provide a high degree of ionization of the created plasma plume, usually with an increased fraction of target metal ions. Surface bombardment with energetic particles leads to the deposition of thin films with a dense microstructure, thus obtaining good properties of coatings, especially higher hardness and corrosion resistance, and a low friction coefficient. Additionally, in comparison to direct current (DC) or radio frequency (RF) magnetron sputtering, HiPIMS can be performed at significantly lower temperatures [8]. The lower temperature not only decreases the cost of the process but allows more materials to be used as a substrate. A deposition process at 400 °C allows hardened steels, nitrided steels, and medium-melting alloys to be used. Using a lower temperature also makes it less difficult to heat bigger elements. In the research presented in this paper, we deposited a tungsten-tantalum diboride coating by means of high-power impulse magnetron sputtering from the single sputtering W-Ta-B target. The influence of pulse duration and bias voltage on the properties of the deposited films is presented and discussed. The novelty of this study is the use of HiPIMS to deposit W-Ta-B coatings with different pulse durations and bias voltages at the fairly low temperature of 400 °C. The mechanical, thermal, and oxidation resistances of the deposited W-Ta-B coatings were evaluated.

## 2. Experimental

### 2.1. Magnetron Sputtering

A cylindrical vacuum chamber (PREVAC, Rogów, Poland) equipped with a turbomolecular pump was used to perform all of the depositions. A single two-inch W_0.76_Ta_0.24_B_2.5_ target prepared by the SPS technique [9] was used for the deposition of all coatings. The vacuum chamber was initially pumped by a turbomolecular and rotary pump down to a pressure of 5 × 10^−7^ mbar. Si (1 0 0) wafers (ITME, Warsaw, Poland) with dimensions of 10 × 10 × 0.7 mm were used. The substrates were mounted parallel to the target surface at a distance of 8 cm on a rotating holder. Prior to deposition, all substrates were heated to 400 °C; this temperature was also maintained during the deposition process. The HiPIMS technique was performed under a pressure of 0.9 Pa, which was controlled by an argon flow during the deposition (10–11 sccm). The parameters related to the HiPIMS hiP-V 6 kW (hiP-V, Holzgerlingen, Germany) power supply are listed in Table 1. Before deposition, the target was sputtered for 10 min with a closed substrate shutter to avoid contamination to a greater extent. The duration of the deposition process was 90 min for all coatings.

### 2.2. Characterization

The surface roughness of the coatings was evaluated with a VK-X100 laser confocal microscope (Keyence, Mechelen, Belgium). The measurements were conducted according to the ISO 4288 standard. Five profiles were measured on each of the tested surfaces. The arithmetic mean roughness value *R_a_* and maximum roughness height *R_z_* were determined. The phase composition of the coatings was evaluated by means of X-ray diffraction (XRD) using a Bruker D8 Discover diffractometer (Bruker, Karlsruhe, Germany). The parameters of this type of XRD analysis have been presented elsewhere [6]. Crystallite size estimation was performed using Sherrer Equation (1), where *τ* is the crystallite size, *K* is a shape factor (a typical value of 0.9 was taken), *λ* is the wavelength of the X-ray (Cu radiation was used, so *λ* = 1.5418 Å), *β* is the FWHM of the selected peak, and θ is the Bragg angle.

(1) $$\tau =\frac{K\lambda }{\beta \cos\theta }$$

The surface of each deposited film was also observed with a scanning electron microscope (SEM) JSM6010PLUS/LV JEOL microscope (JEOL, Akishima, Japan). As tungsten diborides are electrically conductive [9], no additional conductive layer was used. The chemical composition of the coatings was measured by energy dispersive spectroscopy (EDS). During the composition analysis, a 7 kV accelerating voltage was used. The analysis of light (boron) elements is problematic, especially in the presence of heavy (tungsten, tantalum) elements, and so we calibrated the EDS detector with the use of a commercial W_2_B_5_ standard with 99.9% purity (TYR Material, Huizhou, China).

The mechanical properties, the hardness, and Young’s modulus, were determined using a NanoTest Vantage system (Micro Materials, Wrexham, United Kingdom). Measurements were performed on coatings from the “pulse duration series” and “bias voltage series”, and at least 10 indentations at each load were conducted on each sample. The maximum forces during indentation were set to 5, 7, 10, 15, and 20 mN. To avoid the influence of the substrate, only results with a penetration depth of less than 1/10 of the sample thickness were taken into account. The Olivier–Pharr method was used to analyze the load–displacement curves. In addition to the hardness and Young’s modulus, the elastic recovery *W_e_* [10] was also evaluated, which resembles the ratio of elastic energy to the total energy. In addition, the hardness H and effective Young’smodulus E*, ratio H/E* (where E* = E/(1 − v^2^) [11], E is the Young’s modulus, and v is the Poisson’s ratio) parameter was calculated where applicable. 

To study the thermal properties of the manufactured (W,Ta)B_2_ coatings, we annealed selected films for 20 µs at 0 V (grounded) bias voltage. The annealing was performed in a vacuum in the same vacuum chamber as in the deposition process. The duration of the annealing was 60 min for each sample. The temperatures used were 700 °C and 1000 °C. After annealing, another XRD study was performed. The oxidation resistance was evaluated after treatment in a Czylok PRC1800 metallurgical tube furnace (Czylok, Jastrzębie-Zdrój, Poland) with a maximum operating temperature of 1800 °C under normal air pressure with a duration of treatment of 60 min. The temperatures used were 300 °C, 500 °C, and 700 °C. Subsequently, profilometry, XRD, and SEM analyses were performed to examine the surfaces of the coatings.

## 3. Results and Discussion

### 3.1. Surface Roughness

No delamination was observed for any of the deposited coatings. Their thicknesses are presented in Table 2.

These measurements show that by increasing the duration of the pulse, the deposition rate also increases. Similar results have been obtained by others [12]. The low deposition rates from shorter pulses are probably due to the back-attraction of ionized elements from the target. With longer pulses, the amount of metallic ions decreases, whereas the amount of Ar ions increases. Both of these phenomena contribute to a higher deposition rate. Increasing the bias up to 100 V led to an increase in the thickness of the deposited coatings due to a better attraction of ions to the surface. Above 100 V, the thickness of the deposited coating decreased, probably due to re-sputtering by highly accelerated ions. Similar findings were obtained by Xu et al. [13] and Biswas et al. [14]. There is an initial increase in the deposition rate, but after exceeding a certain value of the voltage, the deposition rate begins to decrease. Depending on the process parameters, we were able to obtain deposition rates in the range of 7–20 nm/min (τ = 200 µs, bias 0 V).

The surface roughness of the coatings is shown in Table 2. During the HiPIMS processing, an arcing can lead to ejecting small portions of the target, which can form debris or droplets on the surface. The surface roughness *R_a_* of the deposited coatings is between 60 and 80 nm. These values are higher than the RF-deposited tungsten borides [15], yet HiPIMS-deposited films can still be considered as smooth surfaces. It seems that there are no significant differences in the surface topography between the coatings deposited with varying pulse durations and those deposited with bias voltages.

### 3.2. Phase Composition

**Figure 1:**
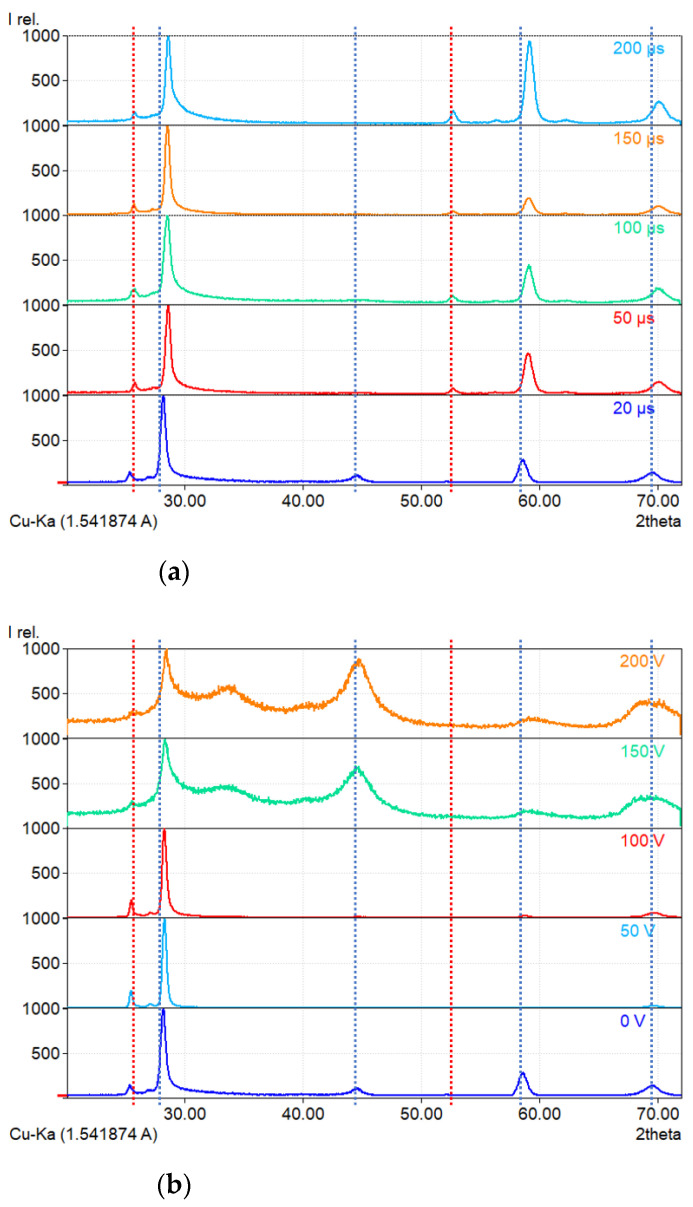
X-ray diffraction patterns of coatings deposited with (**a**) different pulse durations and (**b**) different bias voltages. WB_2_–P6/mmm marked with blue dashed line, WB_2_–P63/mmc marked with a red dashed line.

The X-ray diffraction results are presented in Figure 1. In this figure, Figure 1a shows that the duration of the pulse has no significant effect on the crystallinity of the samples. The detected phases are WB_2_–P6/mmm (peaks at 2θ = 28.1°, 44.6°, 58.6°, and 69.6°) and WB_2_–P63/mmc (peaks at 2θ = 25.3° and 52.7°) [16]. Longer durations of the pulses seem to increase the amount of the P63/mmc phase. In the case of substrate biasing during the HiPIMS process, the XRD results (Figure 1b) show the much greater influence of this parameter during deposition. High voltages (150 V and 200 V) lead to the growth of amorphous/crystalline tungsten diborides. Such a structure can have a significant impact on the mechanical properties of the material.

Based on the Sherrer Equation (1), the crystallite size of the coatings was estimated. All deposited coatings are nanocrystalline (or amorphous/nanocrystalline). The results are listed in Table 3. It should be noted that the shape factor (*K* = 0.9) varies with the shape of the grains. Columnar growth perpendicular to the substrate usually happens during magnetron sputtering, which also happens for tungsten diborides [6,17]. The estimation of the crystallite size for coatings with a high fraction of an amorphous phase can also be greatly affected by the amorphous signal. The small crystallite size in coatings manufactured with 100 µs pulses can be connected with changing fractions of different ions and neutrals during HiPIMS processing. This phenomenon should be further studied by means of emission spectroscopy. In the case of coatings deposited with different bias voltages, they possess similar crystallite sizes. Coatings manufactured using 150 V and 200 V voltages show an amorphous/crystalline structure; therefore, they were excluded from the calculations.

### 3.3. Surface Topography, Chemical Composition

**Figure 2:**
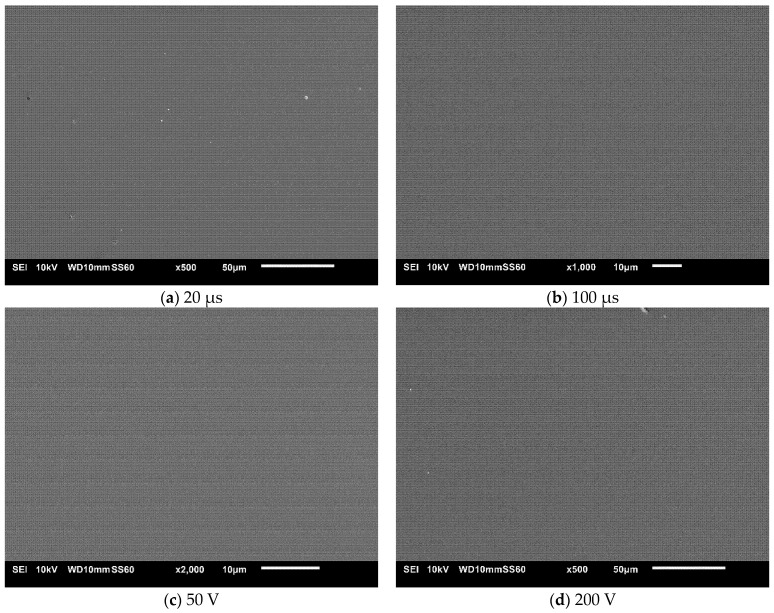
SEM images of selected coatings deposited with a (**a**) 20 µs pulse duration, (**b**) 100 µs pulse duration, (**c**) 50 V bias voltage, and (**d**) 200 V bias voltage.

The surface observation and chemical composition analysis was performed by means of SEM equipped with EDS equipment. As we can see in Figure 2, the surface of the coatings can be described as smooth with randomly placed droplets and debris. Such elements were probably ejected from the target surface during arcing. We expect that by changing the process parameters, it is possible to obtain smoother coatings with fewer defects on the surface. Because of the similarity of the SEM images of the deposited coatings, only four images are presented in Figure 2. Note that different magnifications have been employed.

**Figure 3:**
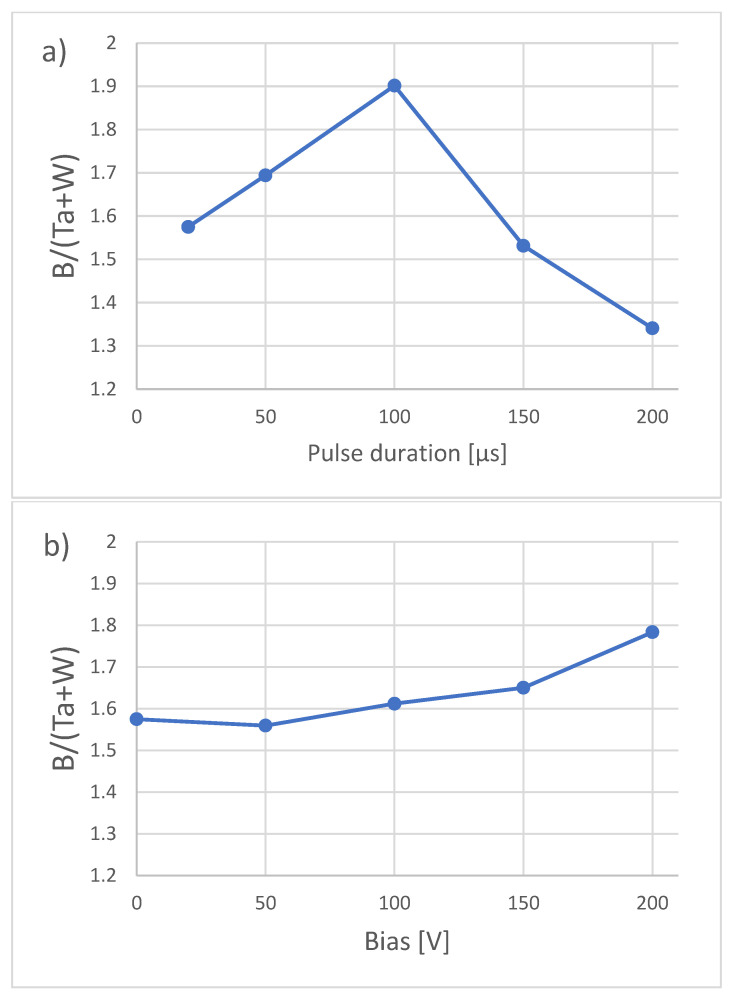
Ratio of boron to metals for different (**a**) pulse durations and (**b**) bias voltages.

EDS analysis of the coatings produced with different pulse durations (presented in Figure 3a) shows that up to the 100 µs pulse duration, the ratio of boron to metals (B/Ta+W) increases with the duration of the pulses, peaking at a value of ≈1.9. Increasing the duration of the pulse from this point to 200 µs yields the opposite tendency, where the ratio falls to a value of ≈1.3.

Even though EDS is an imperfect method for the evaluation of light elements such as boron, such differences are rather significant. Bakhit et al. [18] similarly found that increasing the duration of the pulse up to 80 µs leads to an increase in the boron in the TiB_x_ coatings. Similarly, decreasing the amount of boron ions might be responsible for increasing the boron in the coatings. Fewer boron ions means that less boron will be back-attracted to the target; therefore, more boron will reach the substrate. With longer pulses, the transition from a metallic (and boron) plasma to an argon plasma occurs, and a large number of argon ions can lead to a selective re-sputtering of the coating material. Light elements such as boron are more likely to be re-sputtered than heavy elements such as tungsten and tantalum. This mechanism can be responsible for lowering the amount of boron for longer pulses. The ratio of Ta/Ta+W is very close to the atomic composition of the used target. This is probably due to the fact that the ionic radius, ionization energy, and atomic mass are very similar for tungsten (66 pm, 7.86 eV, 183.84 u) and tantalum (72 pm, 7.55 eV, 180.95 u); therefore, their behavior during discharge in HiPIMS is very similar. The chemical composition of coatings deposited with different bias voltages is shown in Figure 3b. The boron ratio increases with the increase in bias voltage. Previous work carried out in our laboratory by Mościcki [19] shows that boron is easily scattered in a plasma consisting of boron and tungsten. We assume that the scattered ions of boron can be attracted to the substrate by the increased bias voltages, therefore increasing its ratio in the coatings. 

### 3.4. Mechanical Properties

**Figure 4:**
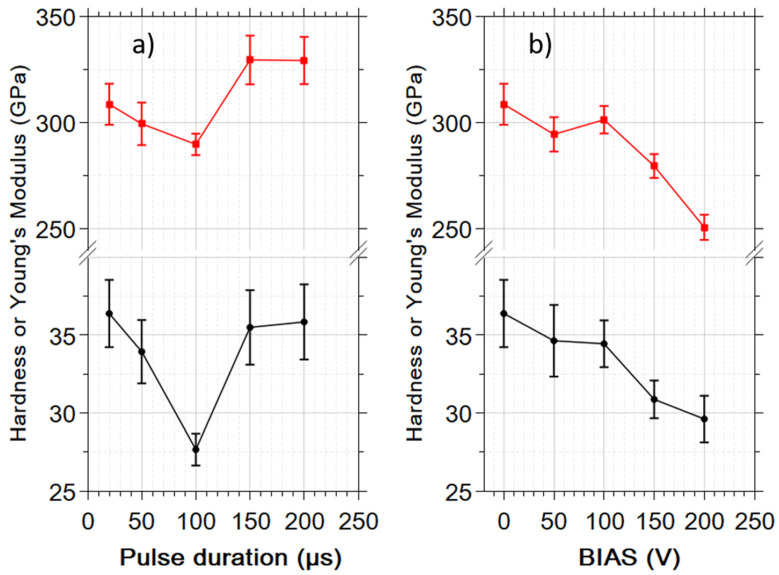
Mechanical properties of coatings deposited with different (**a**) pulse durations and (**b**) bias voltages.

The hardness and Young’s modulus of the deposited coatings are presented in Figure 4. In both cases, the mechanical properties are inversely related to the boron ratio presented in Figure 3. This behavior is consistent with DFT calculations made by Fuger et al. [20]. For WB_2_ that crystallize in a P6/mmm structure (α-type), increasing the boron content lowers the hardness and Young’s modulus. This behavior can probably be explained by vacancy hardening due to dislocation pinning on such defects [21]. Vacancy hardening was also found in tantalum diboride by Šroba et al. [22]. In the case of coatings deposited with different bias voltages, a crucial role in the mechanical properties is played by the amorphous/nanocrystalline composite microstructure. A significant drop in the reduced Young’s modulus occurs in samples that display such a crystal structure. The elastic recovery *W_e_* and H/E* are presented in Table 4. According to Musil [11], H/E* > 0.1 and *W_e_* > 0.6 are usually correlated with good resistance to fracture of coatings.

### 3.5. Thermal Stability and Oxidation Resistance

**Figure 5:**
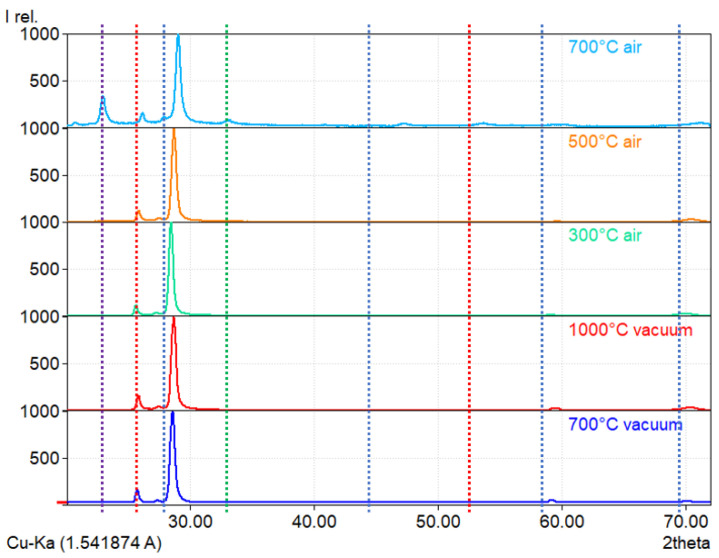
X-ray diffraction patterns of coatings treated in air at 300 °C, 500 °C, and 700 °C and in vacuum at 700 °C and 1000 °C. WB_2_–P6/mmm marked with a blue dashed line, WB_2_–P63/mmc marked with a red dashed line, B_2_O_3_ marked with a green dashed line, and WO_3_ marked with a purple dashed line.

**Figure 6:**
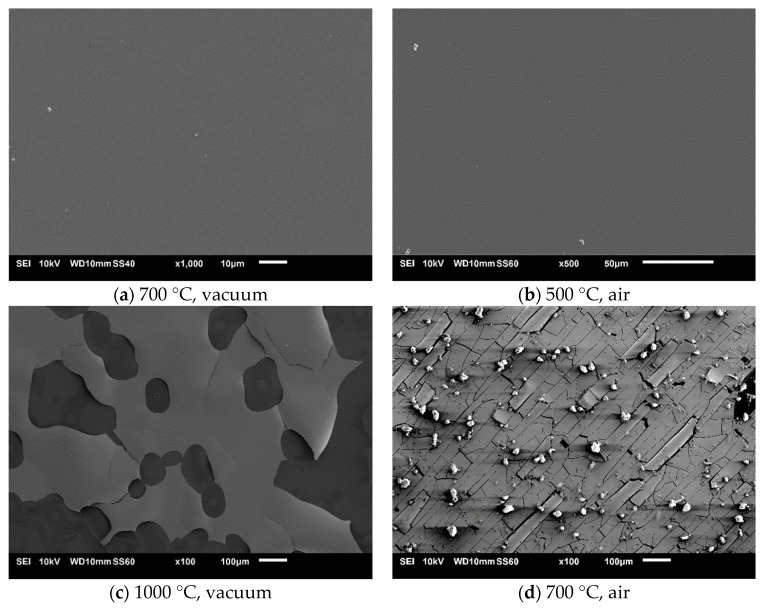

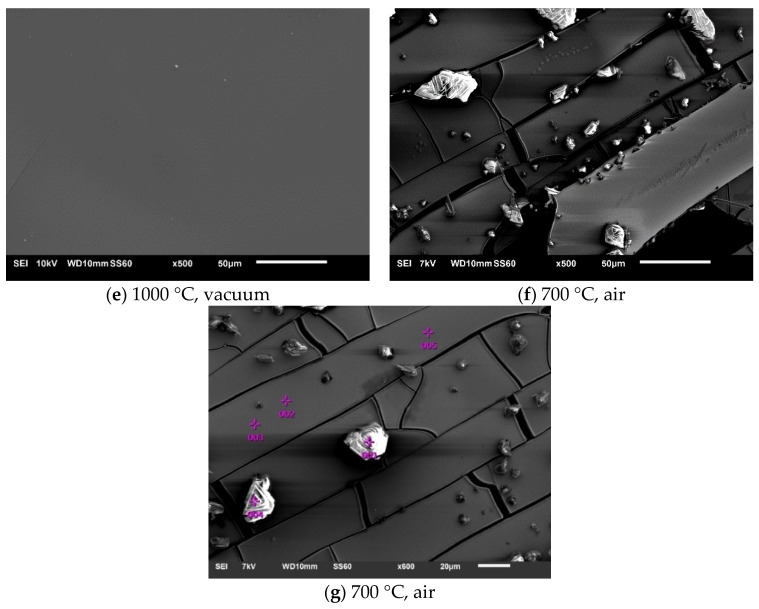
SEM images of coatings treated in vacuum at (**a**) 700 °C and (**c**,**e**) 1000 °C, and in air at (**b**) 500 °C and (**d**,**f**,**g**) 700 °C.

Coatings that annealed in vacuum at 1000 °C and in air at 700 °C were visibly damaged (Figure 6c–f). Both of them were fractured. This probably happened due to a mismatch between the thermal expansion coefficients of the tungsten-tantalum diboride and silicon substrate. Oxidation at 700 °C apparently sped up the fracturing. Despite the visible damage, we were able to obtain a good signal with XRD. The results are presented in Figure 5.

Post-annealed samples show an increasing shift to lower interplanar spacing with the increase in the temperature of annealing. This can be potentially explained by boron diffusing out of the WB_2_ structure. Additional peaks are only present in the coating annealed in air at 700 °C. We identified these peaks as tungsten oxide–WO_3_ (33.1°) and boric oxide–B_2_O_3_ (29.1°). Other peaks can be identified as WB_2_ phases (P6/mmm and P63/mmc). Peaks of these phases were also detected in all previous coatings (Figure 1). This behavior proves that the tungsten diboride alloyed with tantalum is thermally stable at a temperature of at least 1000 °C. Indeed, Moraes et al. [16] found that coatings of (W,Ta)B_2_ can be stable at even higher temperatures. In addition, the literature shows that the presence of boron oxide at the surface may have a positive effect on the wear resistance and coefficient of friction. Peterson et al. [23] showed that at 650 °C, the coefficient of friction of B_2_O_3_ is close to 0.1. For these reasons, we can assume that a tungsten diboride coating alloyed with tantalum can potentially show significant wear resistance, even at high temperatures.

Selected SEM images of samples annealed in vacuum and in air are presented in Figure 6. Coatings treated at 300 °C and 500 °C in air and at 700 °C in vacuum show no significant surface topography changes in comparison to the as-deposited coatings. The coating treated at 1000 °C in vacuum (Figure 6c) was partially delaminated. Magnification of the coating that had not delaminated (Figure 6e) shows that its surface is still rather smooth. EDS analysis in this area shows that there are no distinguishable changes between the coating treated at 1000 °C and the as-deposited coating. Taking into account the XRD results (Figure 5) and EDS analysis, we can conclude that tungsten-tantalum diboride is thermally stable at 1000 °C. Coating delamination probably occurred due to the thermal expansion coefficient mismatch. The image presented in Figure 6d shows significant changes after oxidation at 700 °C. The film is heavily fractured, and some fragments of coating have delaminated and bended (Figure 6f) due to stresses induced by the thermal expansion coefficient mismatch. There is also a massive amount of debris on the surface that might be oxide particles. The chemical composition of points presented in Figure 6g revealed that the bright particles, points 001 and 004 on the image, are composed of boron and oxygen, having 34.32 and 65.68 at.%, respectively. The analysis performed at points 002, 003, and 005 shows the composition of oxygen and tungsten to be 77.05 and 22.95 at.%, respectively. Taking into account the XRD results of the coating annealed in air at 700 °C presented in Figure 5 and the results of EDS analysis, we can confirm the presence of B_2_O_3_ and WO_3_ oxides. Boric oxide has formed particles on the surface, while tungsten trioxide seems to form a layer that resembles the surface of the coating. Such results combined with XRD examination (Figure 5) show that it is highly resistant to oxidation, at least at 500 °C. Similar findings were obtained by Fuger et al. [24], who showed that tantalum has a positive influence on oxidation resistance in tungsten diboride.

## 4. Conclusions

Deposition of hard tungsten-tantalum diboride was successfully performed by using high-power impulse magnetron sputtering (HiPIMS) from one SPSed target. We were able to produce smooth coatings with *R_a_* < 100 nm, where the roughness can be mainly attributed to debris on the surface formed due to arcing. We assume that (W,Ta)B_2_ coatings might be smoother by using different process parameters that can suppress the arcing phenomena. For a pulse duration τ = 200 µs and grounded bias (0 V), quite smooth coatings (*R_a_* = 70 nm) can be produced with a deposition rate of 20 nm/min with the presented setup. For these parameters, the deposited coatings are characterized by very good mechanical properties. They are very hard (37 GPa) and are hypothetically fracture-resistant (H/E* = 0.109, *W_e_* = 0.59). The HiPIMS technique is able to produce crystalline coatings of tungsten diboride at the relatively low temperature of 400 °C. Biasing the substrate can change the structure from crystalline to amorphous/crystalline. Changing the duration of the pulses has a significant effect on the amount of boron in the films. Having a small amount of boron is responsible for obtaining a high hardness of above 37 GPa. Tungsten-tantalum diboride has high thermal stability up to 1000 °C in vacuum (invariance of the crystal phases), and it can withstand oxidation at 500 °C. Coatings with the properties presented in this paper might be good alternatives to materials that are already used in industries.

## Figures and Tables

**Table 1:** Process parameters.

**Pulse Duration Series**	Power	300 W
	Frequency	700 Hz
	Pulse duration	20 µs
		50 µs
		100 µs
		150 µs
		200 µs
	Bias voltage	0 V (grounded)
**Bias Voltage Series**	Power	300 W
	Frequency	700 Hz
	Pulse duration	20 µs
	Bias voltage	0 V (grounded)
		−50 V
		−100 V
		−150 V
		−200 V

**Table 2:** Surface roughness, thickness, and deposition rates of the coatings.

Pulse Duration (μs)	Surface Roughness R_a_	Thickness (μm)	Deposition Rate (nm/min)	Bias Voltage (V)	Surface Roughness R_a_	Thickness (μm)	Deposition Rate (nm/min)
20	0.063	0.95	10.6	0 (grounded)	0.063	0.95	10.6
50	0.067	1.43	15.9	50	0.064	1.14	12.7
100	0.075	1.48	16.4	100	0.065	1.3	14.4
150	0.068	1.64	18.2	150	0.075	1.09	12.1
200	0.07	1.83	20.3	200	0.063	0.88	7.7

**Table 3:** Crystallite size estimation of deposited coatings.

Pulse Duration (µs)	Crystallite Size (nm)	Bias (V)	Crystallite Size (nm)
20	17.7	0	17.7
50	18.4	50	21.8
100	13.5	100	19.8
150	18.6	150	-
200	16.5	200	-

**Table 4:** H/E* and W_e_ of deposited coatings.

Pulse Duration (µs)	H/E*	*W_e_*	Bias Voltage (V)	H/E*	*W_e_*
20	0.118	0.62	0 (grounded)	0.118	0.62
50	0.113	0.62	50	0.118	0.63
100	0.096	0.54	100	0.114	0.63
150	0.108	0.59	150	0.11	0.63
200	0.109	0.59	200	0.118	0.61

## Data Availability

Not applicable.
